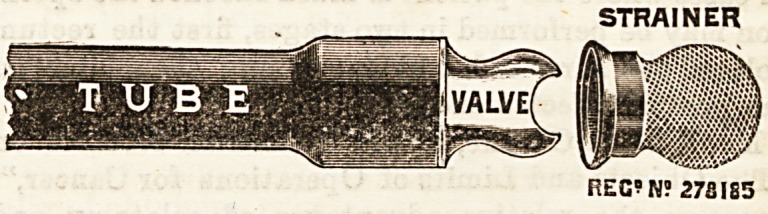# New Appliances and Things Medical

**Published:** 1896-08-08

**Authors:** 


### NEW APPLIANCES AND THINGS MEDICAL.

[We shall be glad to receive, at our Office, 428, Strand, London, W.C., from the manufacturers, specimens of all new preparations and appliances, which may be brought out from time to time.]

### ELECTRIC OZONISERS.

(The Electric Ozone Syndicate, 5, New Union Street, London, E.C.)

The therapeutic effect of ozone must now be seriously reckoned with in the treatment of anæmia, pulmonary tuberculosis, septic conditions of the various passages, and many blood conditions.* Hitherto the chief obstacles to its more extensive adoption in the sphere of practical medicine have been (1) the expense; (2) the difficulty of obtaining it pure. To a large extent these difficulties have been removed by the production of the ozoniser of the above syndicate. The apparatus consists of vacuum tubes containing central metallic cores connected with one of the terminals of a secondary circuit of an inductor coil; the wire connected with the other terminal is wound spirally round the outside of the tubes, and is serrated so as to allow a silent discharge or effluvium. The ozone is formed between the surface of the tubes and the outside wire, and can be collected or allowed to diffuse into the surrounding air. If collected by means of a ball and valve bellows it can be forced through a special mouthpiece and then conveniently inhaled by the patient. It is claimed that either no nitrous compounds or carbonic oxide are formed, or that the proportion is so infinitesimal that it may be neglected. The entire apparatus is put up in a handy form, and can be used in conjunction with the ordinary domestic supply of electricity or with a special battery and induction coil. In either case the entire apparatus costs about £5. It may, however, be hired at a small charge.

* "Nascent Oxygen." The Hospital, July 11th, 1896.

### ENEMA VALVE STRAINER.

(Reynolds and Branson, 13, Briggate, Leeds.)

This is an ingenious but simple contrivance for obviating the blocking of the tail-end valve of the ordinary enema by solid matter or other *débris* contained in the fluid used for injection. It consists of a little wire gauze cap which fits over the free end of the valve, and which acts as a strainer, thus preventing the ingress of larger particles than can conveniently and safely pass through the valve. For giving starch or meal injections its value is obvious, and quite apart from these purposes the accidental blocking of the enema by fluff or other extraneous matter cannot occur, and much trouble and inconvenience thereby is saved.

## Figures and Tables

**Figure f1:**